# Patient-reported adverse effects of high-dose intravenous methylprednisolone treatment: a prospective web-based multi-center study in multiple sclerosis patients with a relapse

**DOI:** 10.1007/s00415-016-8183-3

**Published:** 2016-06-07

**Authors:** Peter Joseph Jongen, Ioanna Stavrakaki, Bernard Voet, Erwin Hoogervorst, Erik van Munster, Wim H. Linssen, Ludovicus G. Sinnige, Wim I. Verhagen, Leo H. Visser, Ruud van der Kruijk, Freek Verheul, Jan Boringa, Marco Heerings, Werner Gladdines, Fredrik Lönnqvist, Pieter Gaillard

**Affiliations:** 1 Department of Community and Occupational Medicine, University Medical Center Groningen, University Groningen, Antonius Deusinglaan 1, 9713 AV Groningen, The Netherlands; 2 MS4 Research Institute, Ubbergseweg 34, 6522 KJ Nijmegen, The Netherlands; 3 to-BBB Technologies BV, J.H. Oortweg 19, 2333 CH Leiden, The Netherlands; 4 Kollwitzstrasse 32, 10405 Berlin, Germany; 5 Department of Neurology, St. Antonius Hospital, P.O. Box 2500, 3430 EM Nieuwegein, The Netherlands; 6 Department of Neurology, Jeroen Bosch Hospital, Henri Dunantstraat 1, 5223 GZ ‘s-Hertogenbosch, The Netherlands; 7 Department of Neurology, Onze Lieve Vrouwe Gasthuis, Jan Tooropstraat 164, 1061 AE Amsterdam, The Netherlands; 8 Multiple Sclerosis Center Leeuwarden, Medical Center Leeuwarden, Henri Dunantweg 2, 8934 AD Leeuwarden, The Netherlands; 9 Department of Neurology, Canisius Wilhelmina Hospital, Weg door Jonkerbos 100, 6532 SZ Nijmegen, The Netherlands; 10 Multiple Sclerosis Center Midden Brabant, ETZ, Location St. Elisabeth, Hilvarenbeekseweg 60, 5022 GC Tilburg, The Netherlands; 11 Department of Neurology, Slingeland Hospital, Kruisbergseweg 25, 7009 BL Doetinchem, The Netherlands; 12 Department of Neurology, Groene Hart Hospital, Bleulandweg 10, 2803 HH Gouda, The Netherlands; 13 Department of Neurology, Meander Medical Centre, Maatweg 3, 3813 TZ Amersfoort, The Netherlands; 14 MH Advies and Organisatiebureau, IJselstraat 81, 9406 TR Assen, The Netherlands; 15 2-BBB Medicines BV, J.H. Oortweg 19, 2333 CH Leiden, The Netherlands

**Keywords:** Multiple sclerosis, High dose, Methylprednisolone, Adverse effect, Side effect, Patient-reported

## Abstract

In a prospective multi-center observational study, we evaluated the frequency, severity, and impact on activities of daily living (ADL) of adverse effects (AEs) of high-dose intravenous methylprednisolone (IVMP) in relapsing remitting multiple sclerosis (MS) patients with a relapse. Online self-report questionnaires stating IVMP’s most common AEs were completed at baseline, the 2nd day of treatment, and 1 day and 1 week after treatment. Eighty-five patients were included, 66 completed the baseline questionnaire, and 59 completed at least one post-baseline questionnaire. Patients reported on average 4 (median) AEs; two (3.4 %) reported no AE. Most frequent was change in taste (61 %), facial flushing (61 %), sick/stomach pain (53 %), sleep disturbance (44 %), appetite change (37 %), agitation (36 %), and behavioral changes (36 %). Of all AEs, 34.3 % were severe and 37.9 % impacted on ADL. A 3-day course resulted in 4 (median) AEs and a 5-day course in 7. All patients with high disease impact had two or more AEs, compared with 79 % of those with low impact (*p* < 0.01). Of patients with high disability, 45 % had severe AEs, compared with 16 % of those with low disability. Severe central nervous system (CNS)-related AEs occurred two times more frequently in patients with high disease impact, and two-and-a-half times more frequently in patients with high disability. Therefore, in virtually all patients, high-dose IVMP leads to AEs, with about one of three AEs being severe with impact on ADL. Patients with high disease impact or high disability may experience more (severe) AEs, due to a higher occurrence of severe CNS-related AEs.

**Electronic supplementary material:**

The online version of this article (doi:10.1007/s00415-016-8183-3) contains supplementary material.

## Introduction

Multiple sclerosis (MS) is a chronic disease of the central nervous system (CNS), in which immune-mediated inflammation and degeneration lead to loss of myelin and axons. In four out of five patients, the disease course is initially characterized by relapses and remissions: relapsing-remitting MS (RRMS) [1]. Most patients fully recover after a relapse, but this can take weeks or months [2]. Treatment with high-dose methylprednisolone shortens the relapse duration and increases the chances of recovery [3]. A European Federation of Neurological Societies task force recommends treatment with intravenous (iv) or oral methylprednisolone in a dose of at least 500 mg daily for 5 days or iv methylprednisolone (IVMP) 1 g daily for 3 days [3].

Methylprednisolone, like other corticosteroids, is associated with a number of adverse effects (AEs), affecting the skin, skeleton, muscles, eyes, CNS, electrolytes, metabolism, and the endocrine, cardiovascular, immune, and gastrointestinal systems, often in a dose-dependent manner [4]. In the USA in 2004, corticosteroids were the most common specific cause for drug-related AEs, accounting for 10.3 % of all drug-related AEs and 141,000 hospital stays [5]. Although most serious AEs are related to the long-term oral use, short-term steroid-induced symptoms are frequent, especially with high-dose treatment needed to treat relapses [6].

Patient-reported outcomes (PROs) receive growing attention in drug research. PROs are measurements of any aspect of a patient’s health status that comes directly from the patient and can be used to evaluate how a treatment affects patients’ functioning and well-being [7]. Studies using PROs to evaluate the AEs of long-term oral corticosteroid treatment showed that patients experience an average of 2.1–2.3 treatment-related symptoms [8, 9].

Most studies on IVMP treatment in MS did not focus on AEs [10, 11]. Despite their frequent occurrence, the severity of IVMP’s AEs is thought to be minor, as they seldom require hospitalization or medical interventions. However, from a patient perspective, this may be questioned, as studies of corticosteroids in general show that they may bother patients and affect the quality of life [9]. In patients with immune thrombocytopenic purpura, AEs of corticosteroids were found to be more bothersome from the patients’ perspectives than from the doctors’ perspectives [9]. Moreover, in MS patients, the distress from CNS-related AEs, like mood change, behavioral change, and sleep disturbance, may add to the burden of MS-related CNS symptoms.

In view of the above, we performed the patient-reported adverse effects of methylprednisolone for relapse treatment in multiple sclerosis (FEEL) study. We specifically assessed from a patient perspective the occurrence, severity, bothering, and impact of AEs during and after high-dose IVMP treatment of an MS relapse. We hypothesized that more (severe) AEs would be reported by patients treated with a 5-day course than by those treated with a 3-day course, and by patients who had not been treated with IVMP in recent years, due to them being less acquainted with IVMP’s AEs. We also expected that CNS-related AEs would be more frequent and more severe in patients with high disease impact and high disability, as we thought it likely that patients with more MS-related CNS dysfunction might be especially susceptible to (severe) AEs affecting the CNS.

## Methods

### Study design and organization

The FEEL study was a prospective, patient-centered, web-based, multi-center study in 15 MS centers and MS specialized neurological practices in The Netherlands. The primary objective was to investigate in patients with RRMS and clinically isolated syndrome (CIS) who were treated with IVMP for a relapse, the frequency, severity, bothering, and impact on activities of daily living (ADL) of AEs; the secondary objective was to investigate the relationship between AEs and the duration of the treatment course (3-day vs. 5-day course), IVMP treatment in the previous two years, and MS-related disease impact and disability. Methylprednisolone was prescribed by the treating neurologist as per regular care and dispensed by the pharmacy as a commercial drug.

The study data were collected using the LimeSurvey software, an open source online application for conducting surveys. Before including the first patient, the MS4 Research Institute’s study platform was extensively tested. Responses were automatically captured. To protect the personal data from unauthorized access, various mechanisms were used to comply with European Union regulations concerning online medical data, including the use of a personal username and a strong password, separation in the database of personal information from the answers to the questions, each screen having a username and password protection, virtual private network tunneling, 256-bits encryption, and the encryption of the participants’ identities via unique 15 digits codes. Automated completeness checks were done before questionnaires could be submitted. The respondents saw an overview of all questions and answers before submission, and they could change the answers before submitting. After submission changes were no longer possible. The help desk (MH) contacted patients by phone in case that they did not succeed in completing the questionnaires. In some cases, questionnaires were completed in paper format at the hospital or at the patients’ home; these were then collected by the site staff, sent to the MS4 Research Institute, and entered by one of the researchers (MH).

### Inclusion procedure and ethical aspects

The inclusion criteria were: (1) diagnosis RRMS or CIS, (2) confirmed relapse, (3) indication for methylprednisolone treatment, (4) willing and able to comply with the protocol, (5) written informed consent, and (6) having access to the internet. During the course of the study, this last criterion was made not applicable, as some of the hospitals preferred to provide the questionnaires in paper form to the patients. The exclusion criteria were: (1) contra-indication for methylprednisolone as defined in the Summary of Product Characteristics, (2) corticosteroid treatment in the previous 30 days, (3) pregnancy or lactation, (4) participation in another study, and (5) progressive MS.

In the study centers, patients with RRMS or CIS who were to start IVMP treatment for a relapse were informed about the study by their treating neurologist or the MS nurse. Patients were informed that they had the right to withdraw consent at any time without prejudice to the neurological treatment or care. After having given their consent patients received a personal code and logged on to the website of the MS4 Research Institute (http://www.ms4ri.nl) to choose a username and password.

The study protocol was presented to the ethical committee *Medisch Ethische Toetsing Onderzoek Patiënten en Proefpersonen* (*METOPP*) (METC nr. M501) in Tilburg, The Netherlands, and the committee concluded that a review was not indicated, as the study did not qualify for being tested according to the Dutch Medical Research Involving Human Subjects Act of 1999 (http://wetten.overheid.nl/BWBR0009408) [12]. The study was performed in agreement with the Declaration of Helsinki (Ethical Principles for Medical Research Involving Human Subjects version 2013; 64th World Medical Association General Assembly, Fortaleza, Brazil, October 2013) (http://www.wma.net) and the *Wet medisch*-*wetenschappelijk onderzoek met mensen* (WMO) (Dutch Medical Research Involving Human Subjects Act) (www.wetten.overheid.nl/BWBR0009408).

The study was financially supported by to-BBB Technologies BV, Leiden, The Netherlands.

### Assessment of adverse effects

AEs were assessed via the Methylprednisolone Adverse Effects Questionnaire (MPAEQ). The MPAEQ inquired about the presence of 15 symptoms that we had previously identified in the literature as being most commonly associated with a short-term methylprednisolone treatment or as very common glucocorticosteroid AEs [3, 9, 10, 13]: facial flushing, feeling sick or having stomach pain, change in taste, change in appetite, sleep disturbance, feeling agitated, feeling angry or bad tempered, feeling depressed, being overoptimistic, behavioral change, muscle weakness, muscle cramps, skin change or delayed wound healing, palpitations, and acne. For each of the 15 items, if an answer was affirmative, then the severity and botheration were quantified (Not at all, A little, Quite a lot, A lot), and the impact on ADL was assessed (Yes, No). Moreover, general questions were asked about the overall health condition (related to MS, to AEs, or both) regarding botheration about the health condition, the health condition’s impact on ADL, and the health condition’s impact on social activities.

The MPAEQ was completed before the start of the IVMP treatment course, at the 2nd day of the treatment, and 1 day and 1 week after the end of the treatment. Therefore, for a 3-day course, the post-baseline assessments were on days 2, 4, and 10, and for a 5-day course, the post-baseline assessments were on days 2, 6, and 12. The baseline MPAEQ was completed at the 1st day of treatment before the first infusion or at the most 2 days earlier. An AE was defined as a new symptom or a worsening of a pre-existing symptom occurring after the start of treatment. This approach enabled the identification of differences between the pre-treatment and follow-up assessments as AEs, and prevented pre-existent symptoms of MS, relapse or concomitant disease from being wrongly associated with the IVMP treatment. An AE was considered severe when the intensity was ‘A lot’ or ‘Quite a lot’ at least once; otherwise, it was mild; an AE was considered bothering when the response to this question was ‘A lot’ or ‘Quite a lot’ at least once. We considered sleep disturbance, feeling agitated, feeling angry or bad tempered, feeling depressed, being overoptimistic and behavioral change as CNS-related AEs, and facial flushing, feeling sick or having stomach pain, change in taste, change in appetite, muscle weakness, muscle cramps, skin change or delayed wound healing, palpitations, and acne as not CNS-related AEs. One day and 1 week after the end of treatment, questions were asked regarding weight increase, infections, use of an iv cannula, premature discontinuation (only 1 week after treatment) and hospitalization (only 1 week after treatment). In addition, the following demographic and disease characteristics were collected at baseline: date of birth, sex, disease modifying treatment, number of relapses, and number of IVMP courses in the last two years.

Neurologists were asked to provide baseline information on the diagnosis, disease course, disease duration, location of treatment, treatment schedule, and the Expanded Disability Status Scale (EDSS) score (optional), and to report any IVMP treatment-related diagnosis 1 week after the end of treatment, using the same study website as the patients.

### Assessment of disease impact and disability

The impact of MS was assessed via the multiple sclerosis impact profile (MSIP) [14, 15]. The MSIP includes 36 questions assessing disability in the domains muscle and movement functions (MMF), excretion and reproductive functions (ERF), basic movement activities (BMA), activities of daily living (ADL), participation in life situations (PLS), environmental factors (EF), and mental functions (MF), and the symptoms fatigue, pain, speech, and vision [14, 15]. The MSIP yields validated domain and symptom scores, where higher scores indicate a worse condition. We considered that the domains MMF, ERF, and MF, and the symptoms speech and vision reflect functions and specific symptoms relating directly to the CNS, and that the domains BMA, ADL, PLS, and EF, and the symptoms fatigue and pain reflect activities and general symptoms that are potentially also influenced by external factors. Therefore, for the purpose of this study, we conceived the MSIP Functions and Specific Symptoms (MSIP–FSS) score, based on the scores of the domains MMF (0–16), ERF (0–12), and MF (0–16), and of the symptoms speech (0–4) and vision (0–4). By use of the formula (MMF*1.25 + ERF*1.67 + MF*1.25 + Speech*5 + Vision*5), the MFIS–FSS score was calculated (minimum 0, maximum 100).

Disability was measured optionally by use of the EDSS [16]. The EDSS is widely used in MS and is based on a neurological examination that provides the basis for the assessment of several functional systems (pyramidal, cerebellar, brainstem, sensory, bowel and bladder, visual, and cerebral or mental) that, according to predefined algorithms, contribute to the EDSS score [16].

### Statistical aspects

No statistical sample size calculation was performed for this observational study. We aimed to include 100 patients, a number that was determined by practical considerations and was considered appropriate to achieve the stated objectives. Descriptive statistics were used for the evaluation of the primary outcomes of this study. In some cases, *p* values were calculated to determine the relevance of observed effects with respect to the secondary outcomes.

## Results

### Patients

**Fig. 1:**
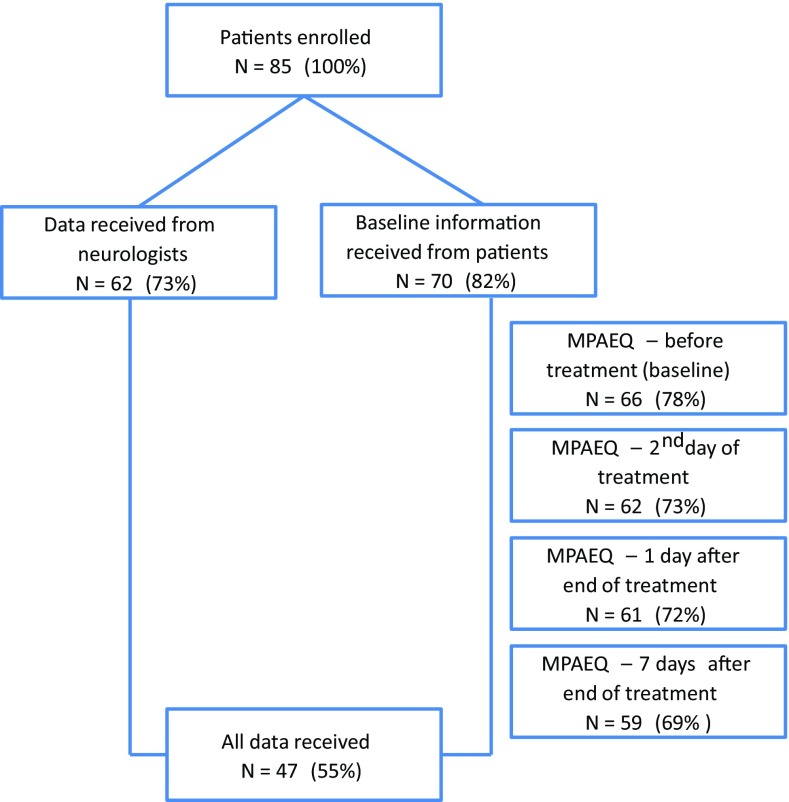
Subject disposition. Numbers (percentages) of patients who were included and who completed the Methylprednisolone Adverse Effects Questionnaire (MPAEQ) at the different time points

Between January 2013 and April 2014, 85 patients were included, i.e., signed the informed consent form (Fig. 1). Seventy (82.4 %) patients provided baseline information on demographic and disease characteristics. Fifty-four (77.1 %) were female, 16 (22.9 %) male; mean [standard deviation (SD)] age was 44.9 (10.9) years (minimum 25, maximum 67). Twenty-nine (41.4 %) patients used disease modifying treatment, 38 (54.3 %) did not [missing 3 (4.3 %)]. Fifty-three (75.7 %) had experienced one or more relapses and 42 (60.0 %) had received one or more IVMP treatment courses during the last 2 years. The MPAEQ was completed at baseline, the 2nd day of treatment, and 1 day and 1 week after the end of treatment by 66 (77.6 %), 62 (72.9 %), 61 (71.8 %), and 59 (69.4 %) patients.

Neurologists provided information on 62 (72.9 %) patients. Fifty-one (82.3 %) patients had RRMS or CIS, and four (6.4 %) progressive MS [information missing in 6 (9.7 %)]. Fifty-two (83.9 %) had a relapse, and four (6.5 %) had no relapse [missing 6 (9.7 %)]. The mean (SD) disease duration was 8.95 (7.48) years (minimum 0.0, maximum 28.0), and the mean (SD) EDSS score (*N* = 32) was 3.0 (1.7) (minimum 0.5, maximum 6.5). Complete patient-reported and neurologist-reported data—with exception of the optional EDSS score—were available in 47 (55.3 %) patients.

### Treatment

IVMP was given in an outpatient clinic, in a hospital and at home in 36 (58.1 %), 18 (29.0 %) and 1 (1.6 %) patient(s), respectively [missing 7 (11.3 %)]. The treatment course was 3 days in 36 (58.1 %) and 5 days in 20 (32.3 %) patients (missing 6 [9.7 %]). The daily IVMP dose was 1000 mg in 49 (79.0 %) and 500 mg in 7 (11.3 %) patients [missing 6 (9.7 %)]. The treatment was completed in 40 (64.5 %) patients and not completed in 1 (1.6 %) [missing 21 (33.9 %)]. The mean (SD) daily IVMP dose (*N* = 56) was 937.5 (166.9) mg (minimum 500, maximum 1000), the mean (SD) total dose (*N* = 56) was 3419.6 (952.4) mg (minimum 1500, maximum 5000), and the mean (SD) durations (per day) of the infusions were between 1.32 (0.48) and 1.44 (0.41) hours (minimum 0.50, maximum 2.50).

### Adverse effects

#### Frequency

**Fig. 2:**
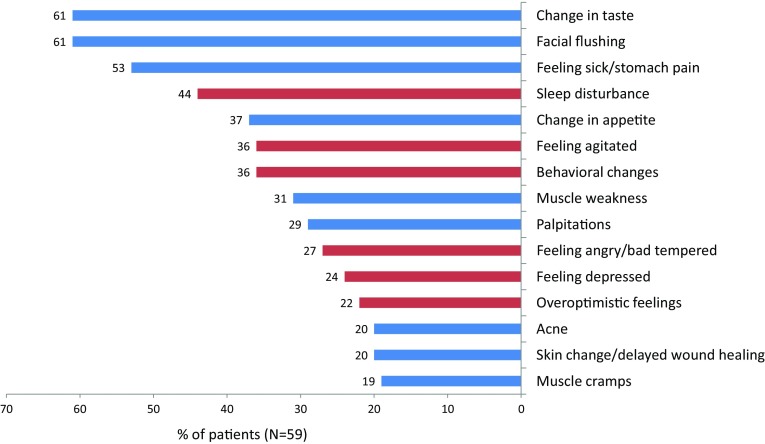
Percentages of patients experiencing a given adverse effect (AE). AEs are marked as CNS-related (*red bar*) and not CNS-related (*blue bar*)

Fifty-nine patients completed the MPAEQ at baseline and at one or more time points after the start of treatment. They reported a total of 306 AEs during and within 1 week after the end of treatment. On average, a patient reported 4 (median) (minimum 0, maximum 12) of the 15 AEs stated in the questionnaire; two (3.4 %) patients reported no AE. The percentages of patients reporting the various AEs are shown in Fig. 2. Most frequent AEs were change in taste (61 %), facial flushing (61 %), feeling sick or having stomach pain (53 %), and sleep disturbance (44 %). The numbers (percentages) of patients reporting AEs at the 2nd day of treatment, and at 1 day and 1 week after the end of treatment, are presented in Table 1.

**Table 1:** Numbers (percentages) of patients who reported adverse effects (AEs), severe AEs, bothering AEs, and AEs with impact on activities of daily living (ADL) at the 2nd day of an intravenous methylprednisolone (IVMP) treatment course, 1 day after the end of the treatment course, and 1 week after the end of the treatment course

	Day 2 IVMP (*N* = 59) (%)	1 day after IVMP (*N* = 58) (%)	1 week after IVMP (*N* = 56) (%)
No AE	13 (22)	4 (7)	16 (29)
One AE	13 (22)	8 (14)	14 (25)
Two or more AEs	33 (56)	46 (79)	26 (46)
Severe AE(s)	46 (30)	60 (29)	41 (33)
Bothering AE(s)	34 (22)	51 (25)	30 (24)
AE(s) with impact on ADL	39 (25)	69 (34)	51 (41)

#### Severity, bothering, and impact on activities of daily living

**Fig. 3:**
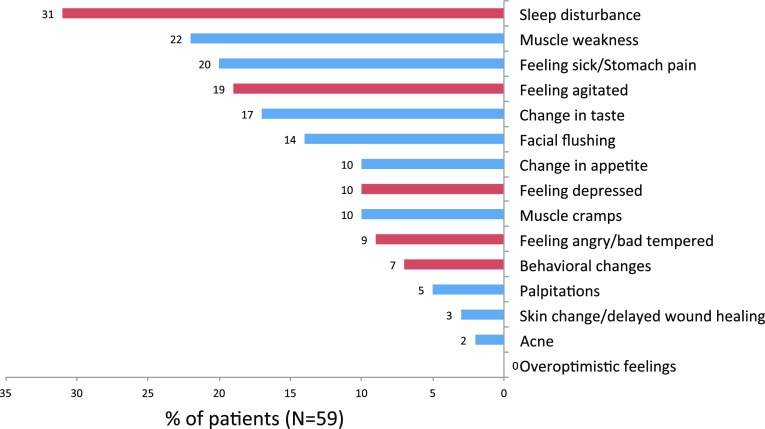
Percentages of patients experiencing a given adverse effect (AE) and considering it severe. AEs are marked as CNS-related (*red bar*) and not CNS-related (*blue bar*)

The numbers (percentages) of patients reporting one or more severe AEs, bothering AEs, and AEs with impact on ADL at the various time points are shown in Table 1. Of all AEs, 34.3 % (*N* = 105) were severe. The percentages of patients reporting a given AE and considering it severe are presented in Fig. 3. Twenty-two (37.3 %) patients had no severe AE, but on average, one (median) (minimum 0, maximum 7) severe AE was reported per patient. The most frequent severe AEs were sleep disturbance (31 %), muscle weakness (22 %), feeling sick or having stomach pain (20 %), and being agitated (19 %). In contrast, overoptimistic feelings (0 %), acne (2 %), and skin change or delayed wound healing (3 %) were rarely severe, also compared with their overall occurrence (22, 20, and 20 %, respectively).

Seventy-nine AEs (25.8 %) were rated as bothering. Thirty (50.8 %) patients reported at least one bothering AE (median 0, minimum 0, maximum 6).

**Fig. 4:**
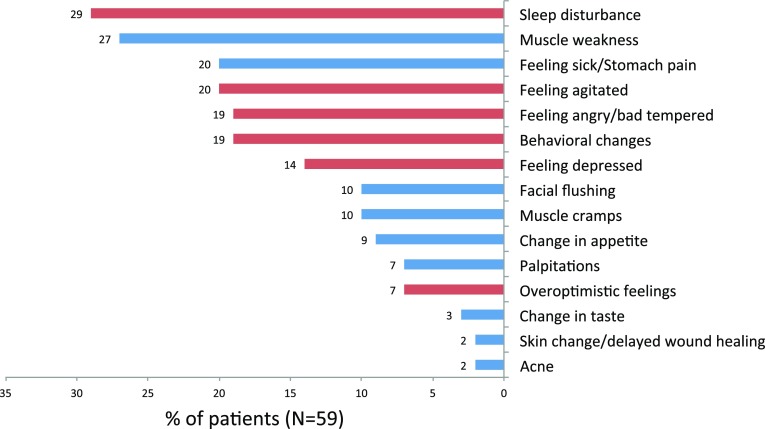
Percentages of patients experiencing a given adverse effect (AE) and stating that it affected their activities of daily living (ADL). AEs are divided into CNS-related (*red bar*) and not CNS-related (*blue bar*)

One-hundred-and-sixteen AEs (37.9 %) had an impact on ADL, and 38 (64.46 %) patients reported at least one AE with an ADL impact (median 1, minimum 0, maximum 8). Figure 4 shows the percentages of patients reporting a given AE that affected their ADL. Sleep disturbance (29 %), muscle weakness (27 %), feeling sick or having stomach pain (20 %), and feeling agitated (20 %) were the most commonly reported AEs that impacted ADL.

#### Other and serious adverse effects

One week after treatment, 17 patients (28.8 %) reported to have gained weight of 1 kg or more and six patients (10.2 %) reported to have suffered an infection (three bladder infections). Five patients (8.5 %) stated that they considered refusing IVMP treatment every time they are treated, and seven (11.9 %) reported to sometimes consider refusing treatment. As much as 57 patients (93.4 %) reported the use of an intravenous cannula during the treatment period, for seven patients (11.4 %), this was bothering and for 21 (34.4 %), it interfered with their ADL.

Neurologists reported to have made an IVMP treatment-related diagnosis in three (4.8 %) patients (acute coronary syndrome, diabetes mellitus, one unspecified) out of 62; however, for 21 patients (33.9 %), no information was provided.

#### 3-day vs. 5-day course

The number of AEs in the 3 days 1000 mg/day group (*N* = 30) was 153, yielding a median of 4 AEs per patient (minimum 1, maximum 11). In the 5 days 1000 mg/day group (*N* = 12), the number of AEs was 85, which yields a median of 7 AEs per patient (minimum 2, maximum 12). The number of severe AEs was 49 (32.0 % of all AEs) and 23 (27.1 % of all AEs) in these two groups, respectively.

#### Treated in previous two years vs. not treated

Comparing the frequencies of (severe) AEs in patients who had been treated with IVMP in the previous 2 years vs. those without such treatment, we found no differences. In the former group (*N* = 34), a total of 176 AEs (median 4 AEs per patient, minimum 0, maximum 12) and 56 (31.8 % of all AEs) severe AEs were observed. The latter group (*N* = 22) experienced 118 AEs (median 5 AEs per patient, minimum 0, maximum 12) and 47 (39.8 % of all AEs) severe AEs.

#### High vs. low disease impact and disability

In patients (*N* = 65) who completed the MSIP, the mean (SD) MSIP–FSS score was 16.80 (9.32) (median 15.42, minimum 1.25, maximum 37.94). We evaluated the MSIP–FSS by exploring the relationship between this patient-reported score on disease impact and the doctor-reported EDSS score on disability. In patients (*N* = 15) with an EDSS score <3.0, the mean (SD) MSIP–FSS score was 12.28 (9.54) (median 10.42, minimum 1.25, maximum 36.68), and in patients (*N* = 12) with an EDSS score ≥3.0, it was 17.89 (9.30) (median 15.43, minimum 9.59, maximum 33.35). The Wilcoxon two-sample test found a relevant difference (*p* = 0.083) between an EDSS score of <3 and ≥3 for the MSIP–FSS score. Moreover, the Spearman correlation coefficient between the MSIP–FSS score and the EDSS score was 0.59, showing a moderate relationship between the two scales.

A relevant difference was found between patients with a low (≤15) and a high (>15) MSIP–FSS score regarding the number of patients with two or more AEs: 78.6 vs. 100 % (*p* < 0.01, Fisher’s exact test) (Table 2). Moreover, patients with a high EDSS score (*N* = 11) reported 21 severe AEs (44.7 % of all AEs), whereas patients with a low EDSS score (*N* = 15) reported 13 severe AEs (15.7 % of all AEs).

**Table 2:** Overview of adverse events (AEs)

	All patients (*N* = 59)	3 days 1000 mg/day (*N* = 30)	5 days 1000 mg/day (*N* = 12)	No previous IVMP course (*N* = 22)	At least one IVMP course (*N* = 34)	EDSS < 3 (*N* = 15)	EDSS ≥ 3 (*N* = 11)	MSIP-FSS ≤ 15 (*N* = 28)	MSIP-FSS > 15 (*N* = 30)
Patients with									
No AE	2 (3.4 %)	0	0	1 (4.5 %)	1 (2.9 %)	0	1 (9.1 %)	2 (7.1 %)	0
One AE	4 (6.8 %)	2 (6.7 %)	0	1 (4.5 %)	3 (8.8 %)	2 (13.3 %)	0	4 (14.3 %)	0
2 or more AEs	53 (89.8 %)	28 (93.3 %)	12 (100 %)	20 (91.0 %)	30 (88.3 %)	13 (86.7 %)	10 (90.9 %)	22 (78.6 %)	30 (100 %)
AEs per patient									
Median	4	4	7	5	4	5	4	4	5
Range	0–12	1–11	2–12	0–12	0–12	1–12	0–9	0–12	2–11
Total number of AEs	306	153	85	118	176	83	47	135	169
Severe AEs	105 (34.3 %)	49 (32.0 %)	23 (27.1 %)	47 (39.8 %)	56 (31.8 %)	13 (15.7 %)	21 (44.7 %)	44 (32.6 %)	61 (36.1 %)
Bothering AEs	79 (25.8 %)	37 (24.2 %)	20 (23.5 %)	33 (28.0 %)	45 (25.6 %)	12 (14.4 %)	15 (31.9 %)	34 (25.2 %)	45 (26.6 %)
AEs with impact on ADL	116 (37.9 %)	52 (34.0 %)	36 (42.4 %)	50 (42.4 %)	66 (37.5 %)	27 (32.5 %)	18 (38.3 %)	51 (37.8 %)	65 (38.5 %)

*IVMP* intravenous methylprednisolone, *EDSS* expanded disability status scale score, *MSIP-FSS* multiple sclerosis impact profile-functions and specific symptoms score

#### Relationship between CNS-related adverse effects and disease impact and disability

With respect to the total number of CNS-related AEs, no differences were found between patients with high vs. low disease impact or disability In contrast, the total number of severe CNS-related AEs in patients (*N* = 30) with high disease impact was 31, whereas the number of severe CNS-related AEs in patients (*N* = 28) with low disease impact was 13 (Additional file). Likewise, the total number of severe CNS-related AEs in patients (*N* = 11) with high disability was 12, whereas the total number of severe CNS-related AEs in patients (*N* = 15) with low disability was 5. Similar differences were not found with respect to severe not CNS-related AEs. For more information about CNS-related vs. not CNS-related AEs with respect to disease impact and disability, see Additional file.

#### Overall health condition: botheration, impact on ADL, and impact on social activities

The data on the botheration about the overall health condition—related to MS, to AEs, or both—and about the health condition’s impact on ADL and social activities suggest that the health condition was highest at the 2nd day of treatment, whereas at 1 day and 1 week after IVMP, it was approximately as low as before treatment (Table 3). It thus seems that the combined burden of MS symptoms and IVMP’s AEs after treatment equals the burden of MS disease during a relapse before IVMP treatment.

**Table 3:** Numbers (percentages) of patients with botheration about multiple sclerosis (MS) symptoms or intravenous methylprednisolone (IVMP) adverse effects (AEs), and of patients experiencing an impact of symptoms or AEs on activities of daily living (ADL) and on social activities, before treatment, at the 2nd day of treatment, and 1 day and 1 week after the end of treatment

MS symptoms, AEs	Baseline (*N* = 66) (%)	Day 2 IVMP (*N* = 62) (%)	1 day after IVMP (*N* = 61) (%)	1 week after IVMP (*N* = 59) (%)
Botheration	27 (40.9)	18 (29.0)	22 (36.1)	23 (39.0)
Impact on ADL	26 (39.4)	18 (29.0)	20 (32.8)	25 (42.4)
Impact on social activities	25 (37.9)	19 (30.6)	21 (34.4)	22 (37.3)

## Discussion

During and within 1 week after high-dose IVMP treatment, patients reported on average five of the most common AEs; only two (3.4 %) patients reported no such AE. Most frequent were change in taste, facial flushing, and feeling sick or having stomach pain. About one-third of the AEs were considered severe, and on average, each patient suffered from one severe AE. The most common severe AEs were sleep disturbance, muscle weakness, feeling sick or having stomach pain, and being agitated, which were reported by one-third to one-fifth of the patients. Also, one out of four AEs were bothersome, one out of three AEs had an impact on ADL, and a longer treatment course, viz. a higher cumulative dose was associated with more AEs.

These observations in daily practice are in line with a recent study in 49 MS patients treated in a randomized controlled trial with iv or oral methylprednisolone for a relapse [10]; all patients except one reported at least one AE, more than half of the patients reported at least one very bothersome AE, and the prevalence of the eight commonly attributed self-reported AEs was significantly associated with the cumulative and average corticosteroid dose [10]. Another randomized, double-blind, controlled study comparing iv with oral methylprednisolone in RRMS patients with a relapse, reported a 97 % frequency of AEs in both groups [17]; the most common AEs reported in the IVMP group where metallic taste (81 %), insomnia (64 %), headache (64 %), hot flashes (59 %), epigastric pain (45 %), and anxiety (37 %), which are similar to those reported by the patients in our study even though a different wording of AEs was used [17].

A finding that to our knowledge has not been reported previously is the association between AEs and disease impact and disability. On further analyses, it seemed that this association may be due to severe CNS-related AEs occurring two times more frequently in patients with high disease impact, and two-and-a-half times more frequently in patients with high disability, than in those with low disease impact and low disability, respectively. In fact, it was one of the study’s hypotheses that patients with more CNS dysfunction, due to the MS disease process, would be more susceptible to methylprednisolone interference with the CNS function. After all, glucocorticoids do influence the function of neurons and microglia [18], and since long corticosteroids have been associated with CNS-related AEs, especially psychological ones [19]. In disease states with excessive endogenous corticosteroid levels, depression, anxiety, and change in behavior are seen in more than half of the patients [20]. Yet, a review on the chronic use of corticosteroids in neuro-oncological patients did not mention CNS-related AEs as frequently occurring [21]. It may be important for doctors to know that higher MS disability and disease impact may be associated with more severe CNS-related AEs, as the impact rate of CNS-related AEs on patients’ ADL is twice that of not CNS-related AEs (57 vs. 27 %).

About one out of three AEs were severe. The percentages of AEs that bothered patients and that impacted on their ADL (26 and 38 %) were of the same magnitude as that of severe AEs, which seems to confirm the clinical relevance of severe AEs. In contrast, Lienert et al. concluded recently that in CIS and MS patients, IVMP therapy was well tolerated without severe side effects. However, their patients received a lower cumulative dose (5 days 500 mg) than our patients. Moreover, patients were apparently not asked to self assess the severity of their AEs. Similarly, Weusten et al. evaluated the side effects of corticosteroid pulse therapy in patients with active rheumatoid arthritis and stated that in most cases the side effects were mild, however, without using a patient-based definition of severe or mild [22].

Our data show that the combined burden of MS-related symptoms and IVMP’s AEs did not substantially differ between baseline and 1 week after treatment. This observation suggests that 1 week after treatment the likely decrease in the burden of MS disease, due to relapse recovery [23], is neutralized by the burden of still existing AEs of the IVMP treatment.

Our study has several limitations, which we like to address here. First, only 66 (77.6 %) of the 85 included patients completed the baseline MPAEQ, and post-baseline data were obtained in 59 of the 66 patients (89.4 %). Possibly patients with more (severe) AEs did not complete a post-baseline questionnaire. Yet, the size of our study group compares relatively well with those in similar studies [10, 24]. Second, we did not include AEs in the questionnaire that have been found by others to be frequently reported by patients, like headache (54 % in Ramo-Tello et al.), dry mouth (42 % in Lienert et al.), and sweating (27 % in Lienert et al.). This may have resulted in an underestimation of the incidence of common (severe) AEs. Third, data on CNS dysfunction were obtained in a limited number of patients (EDSS, *N* = 32) or by means of a not validated overall score (MSIP–FSS) [14, 15]. Fourth, the numbers of patients in the subgroups for analyses of the secondary objective were rather small, which necessitates a cautious interpretation and may stimulate others to reproduce our results. Fifth, in an observational setting, it is challenging to differentiate AEs from symptoms originating from the underlying disease process [19]. Moreover, the hope of a good outcome after steroid treatment or the occurrence of an early improvement may have influenced the report and assessment of AEs. By asking the same predefined questions before, during, and after treatment using a neutral wording, we tried to assess the AEs in a conservative way. Sixth, the study group was less homogeneous than envisaged, as four progressive patients were treated to halt progression; neither does the study inform about AEs in progressive patients with active disease [25].

Given the potential negative impact of IVMP treatment doctors and nurses should counsel patients about possible AEs [26]. However, the information given may be biased or limited in scope. Until recently, the literature on IVMP was dominated by medical diagnoses of rare serious AEs. This is in line with the discrepancy that we observed between the numbers of AEs reported by patients and by doctors: the neurologists reported three IVMP-related diagnoses in patients on whom information was provided (*N* = 41), whereas 306 AEs were reported by the patients (*N* = 59) themselves.

Further clinical research is needed into the effectiveness and the risks associated with methylprednisolone in MS relapses, as this would enable better informed and more precise treatment recommendations [27]. PROs have already enabled a better evaluation of relapse burden from the patient perspective in terms of quality of life and functional ability [28–30]. A similar approach, using PROs to evaluate the burden of AEs, seems equally important, to enable a clinically relevant benefit-risk assessment of short-term corticosteroid treatment, e.g., in our study, one out of five patients (sometimes) considered refusing IVMP treatment because of AEs.

We hope that our study contributes to better informed choices with respect to high-dose IVMP treatment, by quantitatively and comprehensively informing on the occurrence, severity, and impact of the most common AEs from the patient perspective.

## Electronic supplementary material

Below is the link to the electronic supplementary material.

Supplementary material 1 (DOC 37 kb)
