# Supplementary material for: Patient-reported adverse effects of high-dose intravenous methylprednisolone treatment: a prospective web-based multi-center study in multiple sclerosis patients with a relapse
Source: J Neurol. 2016 Jun 7;263:1641–51. doi: 10.1007/s00415-016-8183-3 (PMC4971042; doi:10.1007/s00415-016-8183-3)
Supplement: Supplementary file 1 — Supplementary material 1 (DOC 37 kb) [file 415_2016_8183_MOESM1_ESM.doc]

**Additional file**. Overview of adverse events by relationship with the central nervous system (CNS).

|  | All patients  (N=59) | | EDSS < 3  (N=15) | | EDSS >= 3  (N=11 ) | | MSIP-FSS <= 15  (N=28) | | MSIP-FSS > 15  (N=30) | |
| --- | --- | --- | --- | --- | --- | --- | --- | --- | --- | --- |
|  | CNS-related | not CNS-related | CNS related | not CNS-related | CNS related | not CNS-related | CNS related | not CNS-related | CNS- related | not CNS-related |
| Patients with  No AE  One AE  2 or more AEs | 18 (30.5%)  10 (16.9%)  31 (52.5%) | 3 ( 5.1%)  10 (16.9%)  46 (78.0%) | 5 (33.3%)  0  10 (66.7%) | 1 ( 6.7%)  4 (26.7%)  10 (66.7%) | 2 (18.2%)  4 (36.4%)  5 (45.5%) | 1 ( 9.1%)  2 (18.2%)  8 (72.7%) | 12 (42.9%)  1 ( 3.6%)  15 (53.6%) | 3 (10.7%)  7 (25.0%)  18 (64.3%) | 5 (16.7%)  9 (30.0%)  16(53.3%) | 0  3 (10.0%)  27 (90.0%) |
| AEs per patient  median  range | 2  0 – 6 | 3  0 – 8 | 3  0 – 4 | 3  0 – 8 | 1  0 – 6 | 3  0 – 4 | 2  0 – 5 | 3  0 – 8 | 2  0 – 6 | 3  1 – 7 |
| Total number of AEs | 111 | 195 | 32 | 51 | 21 | 26 | 50 | 85 | 61 | 108 |
| Severe AEs | 44 (39.6%) | 61 (31.2%) | 5 (15.6%) | 8 (15.7%) | 12 (57.1%) | 9 (34.6%) | 13 (26.0%) | 31 (36.5%) | 31(50.8%) | 30 (27.8%) |
| Bothering AEs | 36 (32.4%) | 43 (22.1%) | 5 (15.6%) | 7 (13.7%) | 9 (42.9%) | 6 (23.1%) | 10 (20.0%) | 24 (28.2%) | 26(42.6%) | 19 (17.6%) |
| AEs with impact on ADL | 63 (56.8%) | 53 (27.2%) | 16 (50.0%) | 11 (21.6%) | 12 (57.1%) | 6 (23.1%) | 26 (52.0%) | 25 (29.4%) | 37(60.7%) | 28 (25.9%) |
